# Editorial: Effector functions of therapeutic antibodies

**DOI:** 10.3389/fimmu.2022.1126966

**Published:** 2023-01-12

**Authors:** Peter Boross, Matthias Peipp, Falk Nimmerjahn

**Affiliations:** ^1^ Genmab BV, Utrecht, Netherlands; ^2^ Stem Cell Transplantation and Immunotherapy, Division of Antibody-Based Immunotherapy, Department of Medicine II, Christian Albrechts University Kiel and University Medical Center Schleswig-Holstein, Kiel, Germany; ^3^ Department of Biology, Institute of Genetics, University of Erlangen-Nürnberg, Erlangen, Germany

**Keywords:** antibodies, glycosylation, IgA, effector functions, Fc receptors, complement, checkpoint

Therapeutic antibodies are broadly used in both oncology and autoimmune indications. Currently there are more than hundred antibodies approved by the FDA and many more are in development. Therapeutic antibodies of the human IgG1 subclass with an unmodified Fc portion are increasingly being replaced with engineered antibodies or novel antibody formats. In order to improve the therapeutic index of antibodies, the Fc portion of IgG can be protein- or glyco-engineered (1). Other IgG subclasses or antibody isotypes are also being explored. These modifications are aimed at altering the natural interactions with immune effector functions, such as Fc receptors or the complement system to ultimately improve the efficacy and safety of therapeutic antibodies.

The effector functions induced by the IgG Fc domain of therapeutic antibodies are characterized by complex interactions *in vivo*. The type and magnitude of these interactions depend on a number of factors. However it is not well understood how *in vitro* results, and pre-clinical *in vivo* studies, translate into clinical efficacy and safety. In *in vitro* assays, IgG effector functions are often studied in isolation and the effector functions in pre-clinical mouse models differ from those available in humans. Therefore bridging data from these different model systems is important to enable the use of preclinical results to predict clinical efficacy.

This Research Topic collates eleven articles focusing on the role of the IgG Fc domain for the effector functions of antibodies and highlights different strategies to alter IgG Fc to modify wanted and unwanted biological effects.

In addition to immune checkpoints on adaptive immune cells, targeting similar checkpoints on myeloid cells are also being explored. Chan et al. provides a comprehensive overview of the most prominent example of these, the CD47-SIRPα axis, and describes other potential myeloid checkpoints that could be explored to enhance immunotherapy. IgA is being explored as an alternative isotype instead of IgG for anti-cancer antibodies. As IgA antibodies predominantly engage Polymorphonuclear Leukocytes (PMNs) as effector cells, the authors also discuss the potential advantage of combining IgA antibody therapy with myeloid checkpoint blockade. Baumann et al. show *in vitro* anti-tumor activity with an IgA2 version of the anti-CD38 antibody daratumumab (Dara) in *in vitro* antibody-dependent cell-mediated cytotoxicity (ADCC) and ADCP assays. The activity of the IgA Dara variant could be further boosted by the inhibition of myeloid checkpoints CD47-SIRPα axis.

Zeller et al. analyzed whether dual checkpoint inhibition of the CD47-SIRPα and HLAI/LILRB1 axes in combination with CD20-directed antibodies is a suitable approach to further boost antibody-dependent cellular phagocytosis (ADCP) of lymphoma cells. Analysis of lymphoma cell lines revealed that the ratio of CD20 to HLA class I cell surface molecules determined the sensitivity to ADCP by the combination of rituximab and CD47 antibody. LILRB1 blockade promoted serial engulfment of lymphoma cells and potentiated ADCP but required CD47 co-blockade and the presence of the CD20 antibody. These data provide first *in vitro* evidence that dual checkpoint blockade of CD47 and LILRB1 may be promising to improve antibody therapy of lymphoma patients through enhancing ADCP by macrophages.

Agonistic antibodies that engage co-stimulatory receptors, such as anti-CD40 antibodies, have great promise but their clinical use is complicated by the small therapeutic window. The challenge is to separate the mechanisms that lead to anti-tumor response from those that result in unwanted toxicity. Salomon and Dahan provide an overview of the next generation of anti-CD40 antibodies. These strategies may include Fc engineering for enhanced FcγRIIB binding, intratumoral administration but also the use of bispecific antibodies to restrict CD40 agonism exclusively at the tumor site or specifically to dendritic cells.

For a number of agonistic therapeutic antibodies the IgG Fc part has been shown to be important for therapeutic activity but also play a role in the induction of unwanted side effects. Some of these antibodies are of the IgG4 isotype which is expected to have mild interactions with human FcγRs. Using a deglycosylated version of urelumab, an anti-CD137 antibody, Reitinger et al. showed that abrogating residual FcγR interactions by glycan removal maintains T cell stimulatory activity while ameliorating toxicity. These results suggest that glyco-engineering of IgG4 is an interesting strategy to explore. Furthermore, their data highlights the importance of dosing in order to separate beneficial effects from toxic side effects.

Protein engineering of the IgG Fc is an attractive strategy to enhance or to ameliorate IgG effector functions. Gehlert et al. used the anti-CD19 antibody tafasitamab in which the Fc domain has been modified by two mutations (S239D/I332E) that result in enhanced ADCP and ADCC. The authors have introduced a third mutation, E345K that improves Fc-Fc interactions and increases hexamer formation on the cell membrane resulting in improved complement activation. This mutation was functional, and resulted in improved Complement-dependent Cytotoxicity (CDC) when effector functions were studied in isolation. Importantly, however, in whole blood assays when all effector functions were available, stronger complement activation negatively impacted FcR engagement, highlighting the need for experimental systems where all effector functions can be studies simultaneously.

IgG1 contains one N-linked glycosylation site (Asn-297) and the glycan structure on the IgG has profound functional effects. In particular the presence of core fucose is of high importance, as antibodies with low core fucose are able to trigger stronger FcγR-mediated effector functions. Golay et al. provided a comprehensive overview of the literature on the biological effects of the IgG core fucosylation both in the context of therapeutic antibodies where it can be explored to modulate antibody effector functions and in physiological polyclonal IgG response.

IgG antibodies lacking the penultimate fucose residue are well known to bind with increased affinity to FcγRIIIa. Hatfield et al. now show that in afucosylated sugar moieties galactose residues play an important role in this enhanced binding. By using an enzymatic transglycosylation approach allowing addition of add defined sugar moieties to IgG, they demonstrate that galactosylation at the α6 but not the α3 antennae was responsible for this enhanced binding. The authors further validate their finding by demonstrating that these IgG glycovariants have an enhanced ADCC activity.

In addition to the impact of the sugar moiety attached to the Fc-domain of antibodies it is well established that glycosylation of FcγRs may also impact the IgG-FcγR interaction. For example a specific sugar moiety in FcγRIIIa is critical for detecting IgG glycovariants with low fucose with higher affinity (2). It remained unclear, however, which sugar residues in IgG or FcγRIIIa associated sugar domains have a major impact on this interaction. Van Coillie et al. addressed this important question by generating well-defined libraries of IgG and FcγRIIIa glycosylation variants and demonstrate that afucosylated IgG1 has the highest affinity for oligomannose containing sugar moieties attached to the Asn162 site on FcγRIIIa. Of note, this FcγRIIIa glycovariant seems to be present predominantly on NK cells and much less on monocytes.

Antibodies have become an essential therapeutic tool to treat autoimmune diseases. Thus, antibodies in the form of cytotoxic or immunomodulatory antibodies have become part of established or experimental treatments. Mariottini et al. discuss which antibody-based treatment options are available for patients with multiple sclerosis (MS) and which antibody-dependent effector mechanisms contribute to the therapeutic effect. In addition, they include an in depth comparison of antibody-based versus other treatment options, such as autologous hematopoietic stem cell transplantation.

de Vor et al. studied the potential of monoclonal antibodies against S. epidermidis to induce phagocytic killing by human neutrophils. Different mAbs recognizing Staphylococcal surface components were characterized. To study the immune-activating potential of selected clones, bacteria were opsonized with mAbs in the presence or absence of complement. Activation of the complement system was essential to induce efficient phagocytosis of S. epidermidis. Similar to the study of Gehlert et al. in the context of cancer, complement activation and phagocytic killing of S. epidermidis could be enhanced by Fc-mutations that improve IgG1 hexamerization on cellular surfaces. The authors were able to show that mAbs could greatly enhance phagocytosis of S. epidermidis in neonatal plasma and provide insights that are crucial for optimizing anti-S. epidermidis mAbs as prophylactic agents for neonatal Central line associated bloodstream infections (CLABSI).

Taken together, a collection of original papers, Reviews and Perspective published in this Research Topic highlight recent insights and extend our understanding of the effector functions of human antibodies. We hope that the new insights will contribute to the better understanding of antibody biology and help translate this knowledge into even more effective therapeutic interventions.

## Author contributions

All three Guest-Editors of this Research Topic; PB, FN and MP contributed to the preparation to the present Editorial article. All authors contributed to the article and approved the submitted version.
